# Real-life patient experiences of TTNS in the treatment of overactive bladder syndrome

**DOI:** 10.1177/17562872211041470

**Published:** 2021-08-31

**Authors:** Manon te Dorsthorst, Michael van Balken, Dick Janssen, John Heesakkers, Frank Martens

**Affiliations:** Radboudumc, Geert Grooteplein Zuid 10, Nijmegen, 6500 HB, Netherlands; Rijnstate, Arnhem, Gelderland, Netherlands; Radboudumc, Nijmegen, Netherlands; Maastricht University Medical Centre, Maastricht, Netherlands; Radboudumc, Nijmegen, Netherlands

**Keywords:** Overactive Bladder syndrome (OAB), Percutaneous Tibial Nerve Stimulation (PTNS), Transcutaneous Tibial Nerve Stimulation (TTNS), urge, urge-incontinence, neuromodulation

## Abstract

**Introduction and objectives:**

Overactive bladder syndrome (OAB) is defined as urinary urgency, with or without urgent urinary incontinence; it is often associated with urinary frequency and nocturia, in the absence of any pathological or metabolic conditions that may cause or mimic OAB. The aim of this study was to evaluate the long-term real-life adherence of transcutaneous tibial nerve stimulation (TTNS) in the treatment of OAB, patient satisfaction of the treatment, and reasons for quitting therapy.

**Materials and methods:**

In this single center study, all patients who had a positive effect on percutaneous tibial nerve stimulation (PTNS) and continued to receive home-based treatment with TTNS since 2012 were included for analysis. Patients were retrospectively asked to fill out a questionnaire regarding satisfaction, reasons for quitting, and additional or next line of therapy.

**Results:**

We included 42 patients for this study, 81% of these patients were female (*n* = 34). The median age was 67 years (range 36–86). Most of the patients (64%, *n* = 27) were diagnosed with OAB wet. The median TTNS treatment persistence was 16 months (range 1–112 months). Reasons and percentages for stopping therapy were: 55% stopped treatment due to loss of effect, and 24% stopped because of preferring other type of neuromodulation. The mean satisfaction score (scale 1–10) in patients who continued TTNS was 6.2 (*n* = 9, SD 1.30) *versus* 5.4 (*n* = 29, SD 2.24) for patients who quit therapy. We did not find a statistically significant difference between the two groups (*p* = 0.174).

**Conclusion:**

TTNS, although effective in the short-term, is not effective in the long-term. In combination with a low satisfaction rate among patients, there is a need for improvement in terms of OAB treatment modalities.

## Introduction

Overactive bladder syndrome (OAB) is defined by the International Continence Society (ICS) as urinary urgency, with or without urgent urinary incontinence, and often associated with urinary frequency and nocturia, in the absence of any pathological or metabolic conditions that may cause or mimic OAB.^1^ OAB treatment starts, according to guidelines, with behavioral therapy and if needed, is supplemented with drug treatment. When drug treatment is unsuccessful, the next line of treatment consists of percutaneous tibial nerve stimulation (PTNS) or intravesical Onabotulinum Toxin A injections or sacral nerve stimulation (SNS).^2^ Success rates of PTNS were described by Peters *et al.*^3^ in the first sham-controlled trial for PTNS. They described an 43% improvement for urinary urgency, 48% improvement for urinary frequency, 38% improvement for urge incontinence (UUI), and an overall improvement of 55%, based on the global response assessment (GRA) scale after 13 weeks of treatment. However, one of the main disadvantages of PTNS is the fact that patients have to come to hospital for their treatment. Secondly, once PTNS treatment is quitted, patient’s complaints will return; therefore, patients will require maintenance therapy.^4^

Home-based treatment with transcutaneous stimulation of the tibial nerve (TTNS) could solve these problems. TTNS uses a surface electrode, instead of a needle, to stimulate the tibial nerve, which can be self-applied by patients.^5^ Previous studies showed that TTNS in the study context is an effective treatment option in the treatment of idiopathic OAB.^5–8^ In particular, Ramírez-García *et al*.^8^ showed non-inferiority in the decrease of daytime frequency voiding in patients with idiopathic OAB and Detrusor Overactivity (DO) in their randomized controlled trial comparing PTNS *versus* TTNS. Both techniques improve symptoms and to a large extent, quality of life (QoL). Moreover, the perception of improvement did not differ between PTNS and TTNS. However, real-life data related to efficacy and continuation of the treatment over the longer term are scarce.

The aim of this study was to evaluate the long-term, real-life adherence of patients to TTNS for the treatment of OAB, including patient satisfaction and reasons for stopping TTNS.

## Material and methods

In this single center study, all patients who had positive effect on PTNS and who continued to receive home-based treatment with TTNS since 2012 were included. Patients were retrospectively asked to fill out a questionnaire (supplementary data) regarding satisfaction, reasons for quitting, and additional or next line therapy. All patients were included in our single center university hospital. All patients started with at least 7 treatment sessions of PTNS (median 18, range 7–49 weeks) once a week, followed by an evaluation session with their urologist. If there was a subjective improvement of their OAB complaints, patients were asked to continue to receive home-based TTNS treatment. Instructions for home-based TTNS were given by a specialized nurse and evaluation was performed two weeks after the start of TTNS therapy. During this follow up moment, it was evaluated if they required additional training. After this, patients continued to receive maintenance therapy with TTNS at home, led by themselves, without any specific follow up.

Inclusions criteria for this study were patients with PTNS followed by TTNS. All patients who started PTNS and/or TTNS under the age of 18 were excluded for analysis, as were patients with mental or physical limitations for filling out the questionnaire [i.e., Alzheimer’s, post cerebrovascular accident (CVA) with physical limitations, illiterate]. Before the questionnaires were sent, patients were called to participate in the study. When patients could not be reached, they were defined as lost to follow up.

Baseline criteria and extra study details were retrieved from patient electronical files after receiving their informed consent. The questionnaire, which was sent to all patients, is included in the supplementary data. Statistical analysis was performed by using SPSS 22.0 (SPSS, Chicago, IL). Kaplan–Meier curves were used to estimate the survival of TTNS. Discontinuation of TTNS was used as an endpoint. The T-test was performed to determine statistical significance between the mean satisfaction score (scale 1–10) of both patient groups: patients who continued the treatment *versus* patients who quitted treatment. This study was approved by the Ethics Committee of the Radboud University Nijmegen Medical Centre (2020-7035). Written informed consent was obtained from all patients for the use of clinical data in research.

## Results

### Baseline criteria

**Figure 1.**
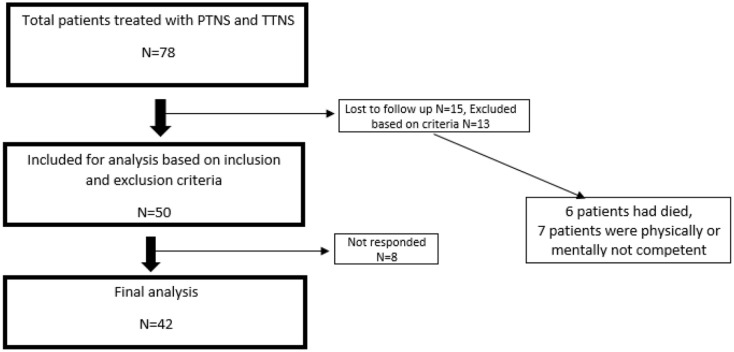
Study flow, patient inclusion/exclusion and number of patients final analysis.

A total of 78 patients underwent PTNS followed by TTNS in our University Medical Center. Questionnaires were sent to 50 patients. The reasons and numbers for not being included in the study were as follows: 15 patients were lost to follow up, 6 patients had died, and 7 patients were determined to not be physically or mentally competent to fill out the questionnaire. Out of our 50 patients who met the inclusion and exclusion criteria, 8 patients did not respond to our request to fill out the questionnaires. As a result, we included 42 patients for this study (response rate intention to treat 55%, response rate per protocol 84%). Figure 1 shows the patient flow.

42 Patients were included; of these, 81% were female (*n* = 34). The median age was 67 years (range 36–86). Most of the patients (64%, *n* = 27) were diagnosed with OAB wet, followed by OAB dry (19%, *n* = 8) and neurogenic origin (17% *n* = 7). A total of 67% (*n* = 28) of the included patients had previous medication and pelvic floor therapy, while 31% (*n* = 13) had medication only. For one patient it was unclear what treatment was received prior to the PTNS.

### PTNS analysis

All patients received weekly PTNS sessions prior to their treatment with TTNS. The median duration of their PTNS treatment was 18 weeks (range 7–49 weeks). We could not determine the duration of treatment for 3 patients, as these patients were treated in a hospital near their home instead of at our referral center.

### TTNS analysis

**Figure 2.**
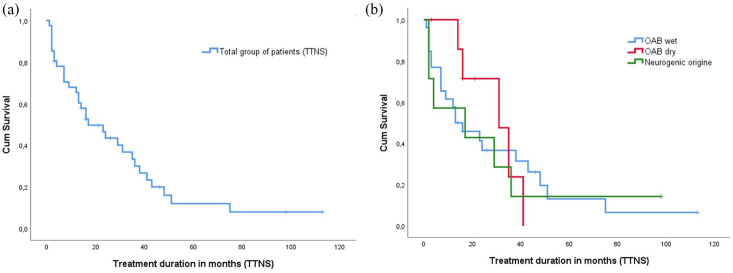
(a) Treatment duration of TTNS in months among all patients (*n* = 42) (b) Treatment duration of TTNS in months specified per category (OAB wet *n* = 26, OAB dry *n* =* 8*, neurogenic *n* = 7). OAB, overactive bladder syndrome; TTNS, transcutaneous stimulation of the tibial nerve.

**Figure 3.**
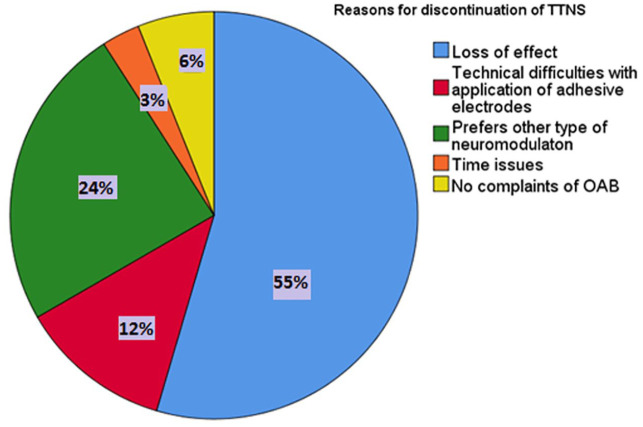
Reasons for discontinuation of TTNS (*N* = 33). TTNS, transcutaneous stimulation of the tibial nerve

All patients continued with TTNS after their PTNS treatment. The median TTNS treatment persistence was 16 months (ranges 1–112 months). Figure 2(a) illustrates the overall survival (OS) of TTNS treatment in all patients. Figure 2(b) illustrates the survival per category of OAB (OAB wet, OAB dry, and neurogenic origin). Unfortunately, due to the low numbers we could not perform any other statistical analysis. During treatment, 45% of the patients used TTNS on a daily basis, followed by 27.5% of the patients who were using it 3–6 times a week, and 22.5% who were using the system 1–2 times per week. Only a small percentage, 5%, used it less than once a week. Twenty-one percent continued treatment; reasons for and percentages for quitting therapy are shown in Figure 3. The main reason to discontinue TTNS was a loss of effect (55%).

During TTNS treatment, almost 62% of the patients did not use any other form of treatment, 36% of the patients used medication as additional therapy, and in 2% it was unknown. The mean satisfaction score (scale 1–10), for which all patients rated their TTNS treatment, was 5.6 [*n* = 38, standard deviation (SD) 2.07]. The mean treatment score in patients who continued TTNS was 6.2 (*n* = 9, SD 1.30) *versus* 5.4 (*n* = 29, SD 2.24) for patients who quit therapy. We did not find a statistically significant difference between the two groups (*p* = 0.174). If patients did stop TTNS, one third did not receive or continued with a different OAB treatment. When they opted for a different treatment, they mainly choose PTNS (21%), PTNS implant (18%), botox (12%), medication (9%), or sacral neuromodulation (6%).

## Discussion

We report that, in this real-life study, the median treatment persistence of TTNS is only 16 months after start treatment. In addition, 55% of patients quit their therapy because of a loss of effect over time, while most of them were treating themselves on a daily base. These results illustrate the main short comings of TTNS over time. TTNS has a positive effect on OAB symptoms; this is shown for short term follow up by Booth in their sham controlled TTNS study, and Schreiner in their randomized controlled trial (RCT), where patients received TTNS in addition to standard therapy [bladder training, programme of repeated voluntary pelvic floor muscle contraction (PFMT)].^9,10^ Although TTNS is successful at the start, we observed that most patients do not continue with their treatment in the long term due to the numerous reasons provided above.

If we compare our real-life data regarding the long-term treatment efficacy to existing studies, we do see slightly different outcomes. Leroux *et al.*^7^ describe in their study a mean TTNS persistence of 8.3 months: 29% of their patients continued for over 12 months. Only 16% continued for 18 months or more, compared to our median follow up of 16 months. Only 17% of their patients continued treatment with TTNS during their end of study moment; on contrast, we found this figure to be 21%. The reasons for this difference could be explained by the number of patients included in both studies, but also by the differences in design of both studies. However, it can be concluded that the results from both studies show that TTNS treatment persistence is currently not satisfactory.

Comparing our long-term TTNS therapy adherence data to real-life PTNS data, we do not see a large difference in outcome. As previously discussed, the median treatment persistence in our study was 16 months (*n* = 42). In real-life PTNS studies (*n* = 183) the median follow up of patients during maintenance treatment is 18 months.^11^ Sirls *et al.*^12^ describe in their real-world study that 55% of their patients continued maintenance PTNS treatment after 3 months. However, the reasons for discontinuation of PTNS differ from TTNS, mainly because of the logistic intensity (frequent clinical visits) of the PTNS treatment compared to TTNS.^11^

The main reason for TTNS discontinuation was a loss of efficacy or a lack of sufficient symptom relief. This is in line with other publications. Leroux describes that 70% of their patients stopped treatment due to a lack of sufficient symptom relief.^7^ They further mentioned compliance difficulty and becoming asymptomatic as reasons for discontinuation. This could be the reason why, in their series, 70% of patients experienced a loss of effect; this is higher compared to the 55% in our study.

In this study we could only quantify the level of satisfaction rate of patients by recall for both treatment periods with PTNS and TTNS. Patients rated their TTNS treatment generally, with an overall 5.6 (scale 1–10) report grade. This fairly low score, in addition to the fact that patients often preferred a different form of TNM (24%) suggests that other forms of TNM had more satisfactory outcomes. These observations are in contrast to a RCT published by Martin Garcia followed by a non-inferiority study by Ramírez-García.^6,8^ Both studies concluded that there was no statistical difference in efficacy outcome and QoL questionnaires in TTNS *versus* PTNS.

Posterior tibial neuromodulation, and as a part of this TTNS, has proven its efficacy over the years in the treatment of OAB.^3,9,13^ However, similar to other OAB treatment modalities, long term therapy adherence is poor and alternative treatment options are scarce. For the most part, OAB patients stop these forms of therapy because of side effects or a lack of efficacy in the longer-term.^11,14^ In our view, this is also the case for TTNS in the real-life setting. The limitations of our study are the number of patients which were included and the single center nature of the study. As shown in Figure 2(b), there could be some differences between OAB categories. However, our numbers were too low and this poses a limitation on reaching a solid conclusion. We hope that a more patient tailored, minimally-invasive treatment modality could enhance persistence and adherence for patients utilizing the current OAB treatment modalities in the future. As a result, developments in the efficacy of tibial nerve stimulation utilizing implantable devices is, therefore, of interest.^15–17^

## Conclusion

Although many publications report a positive effect of TTNS on patients suffering from OAB in short term follow up, TTNS in the long term is not that effective in real-life. In combination with a low satisfaction rate, the need for other OAB treatments is still persistent. In order to appreciate the value of treatment modalities, also for OAB, more research in the real-world setting is needed.

## Supplemental Material

sj-pdf-1-tau-10.1177_17562872211041470 – Supplemental material for Real-life patient experiences of TTNS in the treatment of overactive bladder syndrome Click here for additional data file. Supplemental material, sj-pdf-1-tau-10.1177_17562872211041470 for Real-life patient experiences of TTNS in the treatment of overactive bladder syndrome by Manon te Dorsthorst, Michael van Balken, Dick Janssen, John Heesakkers and Frank Martens in Therapeutic Advances in Urology
