# Supplementary material for: Real-life patient experiences of TTNS in the treatment of overactive bladder syndrome
Source: Ther Adv Urol. 2021 Aug 31;13:17562872211041470. doi: 10.1177/17562872211041470 (PMC8411642; doi:10.1177/17562872211041470)
Supplement: sj-pdf-1-tau-10.1177_17562872211041470 – Supplemental material for Real-life patient experiences of TTNS in the treatment of overactive bladder syndrome [file sj-pdf-1-tau-10.1177_17562872211041470.pdf]

Studie ID: …………………………………………….

Vragenlijst

Wij willen u vragen onderstaande vragenlijst <u>correct</u> en <u>volledig</u> in te vullen. Nadien graag retour sturen middels bijgevoegde retourenvelop.

1. Gebruikt u op dit moment nog de zenuwstimulatie met plakkers op uw enkel te plakken?

- □ **Ja, ga verder bij vraag nr 2 t/m 4**
- □ **Nee, ga verder bij vraag nr 5 t/m 10**

<u>Indien Ja:</u>

2. Hoe vaak gebruikt u deze behandeling nog op dit moment?

- □ Dagelijks
- □ 3-6 maal per week
- □ 1-2 per week
- □ Maandelijks
- □ Anders namelijk…..

3. Bent u tevreden over de behandeling van uw plasklachten met de thuisstimulatie door plakkers? (Schaal 1-10, zeer ontevreden 1-2-3-4-5-6-7-8-9-10 zeer tevreden)

………………………………………. punten

4. Is er naast uw thuisstimulatie nog een andere behandeling gestart voor uw plasklachten?

- □ Nee
- □ Ja, fysiotherapie gericht op de plasklachten
- □ Ja, medicatie gericht op de plasklachten
- □ Ja, alternatieve geneeswijze gericht op de plasklachten
- □ Ja, anders…………………………..

<u>Indien Nee:</u>

5. Sinds wanneer bent u gestopt met de thuisstimulatie met plakkers voor uw plasklachten?

Gestopt omstreeks (maand/ jaartal): ………………………………………………
□ Weet niet

6. Hoe lang heeft u uzelf in totaal behandeld door middel van thuisstimulatie met plakkers voor uw plasklachten?

………….. maanden / jaren (doorhalen wat niet van toepassing is)

7. Hoe vaak gebruikte u de thuisstimulatie voor uw plasklachten nog op het moment dat u uzelf nog behandelde?

□ Dagelijks
□ 3-6 maal per week
□ 1-2 per week
□ Maandelijks
□ Minder dan 1x per maand
□ Anders namelijk…..

8. Wat was de reden van stoppen van de behandeling?

□ Moeilijkheden met plaatsen van de plakkers
□ TENS apparaat is kapot gegaan
□ Voorkeur voor andere behandeling
□ Geen effect meer waarneembaar ondanks dat plakkers goed zitten en kastje werkt.
□ Geen mogelijkheid meer qua tijd om behandeling uit te voeren
□ Anders namelijk…………………………………………………….

9. Hoe tevreden was u over de behandeling van uw plasklachten met de thuisstimulatie door plakkers ten tijde van staken van de behandeling? (Schaal 1-10, zeer ontevreden 1-2-3-4-5-6-7-8-9-10 zeer tevreden)

………………………………………. punten

10. Welke behandeling bent u gestart na het staken van de thuisstimulatie door plakkers voor uw plasklachten?

□ Medicatie
□ Botox injecties in de blaas
□ Terug naar enkelstimulatie door middel van naaldjes in het ziekenhuis (PTNS)
□ Sacrale neuromodulatie (stimulatie met kastje in de bilregio)
□ Fysiotherapie
□ Alternatieve geneeswijze
□ Geen behandeling meer gestart
□ Anders namelijk…………………………………………………….
